# A case-control association study of K121Q and G/T Variants in *ENPP1* and *TCF7L2* gene with type 2 diabetes mellitus in North Indian Punjabi Population

**DOI:** 10.1186/1755-8166-7-S1-P115

**Published:** 2014-01-21

**Authors:** Basanti Barna, Badaruddoza, Kawaljit Matharoo, Amarjeet Singh Bhanwer

**Affiliations:** 1 Department of Human Genetics, Guru Nanak Dev University, Amritsar-143005, Punjab, India

## Background

The *K121Q* and G/T polymorphism of the *ENPP1* and *TCF7L2* genes respectively plays a significant role and found to be associated with increased risk of T2DM in many worldwide populations. However, a very few studies have been done for this association in North Indian Punjabi populations. In the present cross section study K121Q polymorphism of *ENPP1* gene and G/T polymorphism of *TCF7L2* gene were analysed to determine the association of these genes with type 2 diabetic North Indian Punjabi subjects.

## Materials and methods

A total of 450 participants consisting of 239 T2DM and 211 healthy subjects were recruited for this study. Genomic DNA was amplified by PCR-RFLP method. Anthropometric and physiometric variables such as height, weight, Body Mass Index (BMI), Waist hip ratio (WHR), Waist circumference (WC), Hip circumference (HC), biceps skinfold, triceps skinfold, SBP, DBP, MBP, Pulse rate and pulse pressure were measured using standard protocol. The Chi-square analyses were used to test the significance difference in genotype, allele frequencies and Hardy-Weinberg equilibrium deviation. Associations of genotypes with T2DM and its corresponding Pvalues were calculated using Web-Assotest program.

## Results

The results revealed that anthropometric and clinical characteristics such as WHR, fasting and random glucose levels, SBP, DBP, pulse rate and pulse pressure have significant (p<0.001) differences between T2DM and control subjects. However, none of the anthropometric and clinical characteristics have found significant difference with respect to their genotypic distributions for both of genes except DBP for *ENPP1* K121Q polymorphism.

## Conclusions

The association analysis of the present results showed K121Q variant of *ENPP1* gene G/T variant of *TCF7L2* gene have significant association with type 2 diabetes with dominant and recessive model of action respectively in North Indian Punjabi populations.

